# Experimental and Simulation Investigation of Octadecyltriethoxysilane-Decorated Diatomaceous Earth Coatings with Enhanced Superhydrophobic and Self-Cleaning Properties

**DOI:** 10.3390/ma18174209

**Published:** 2025-09-08

**Authors:** Aijia Zhang, Nan Xiao, Kunjie Yuan, Wenbin Cao

**Affiliations:** 1 School of Materials Science and Engineering, University of Science and Technology Beijing, Beijing 100083, China; 2 School of Energy and Environmental Engineering, Hebei University of Technology, Tianjin 300401, China; 3 Tianjin College, University of Science and Technology Beijing, Tianjin 301830, China

**Keywords:** diatomaceous earth, superhydrophobicity, self-cleaning, application stability, molecular dynamics

## Abstract

In this study, an effective diatomaceous earth (Dia)/octadecyltriethoxysilane (OTS)/epoxy resin (EP) with enhanced superhydrophobic and self-cleaning coating was prepared by spraying method, and the effect of OTS modification on the hydrophobicity of Dia materials was investigated through molecular dynamics computational simulation. The results showed that the number of hydrogen bonds and electrostatic interaction energy between diatomite and water molecules were significantly reduced after OTS modification, which significantly enhanced the hydrophobicity of diatomite. The coating exhibits excellent superhydrophobic properties, with a contact angle of up to 152.3°, and has a wide range of applicability, being able to uniformly cover a wide range of substrate surfaces such as glass, wood, and aluminium panels. In addition, it demonstrates excellent self-cleaning capabilities, effectively removing surface contaminants. The mechanical and chemical stability of the coating has also been thoroughly investigated, and it remains superhydrophobic even after abrasion tests and shows excellent stability in acidic or alkaline corrosive environments. Molecular dynamics calculations further elucidated the reason for the change in hydrophobicity of the coatings in acidic and alkaline environments, revealing that the diffusion of water molecules slows down in alkaline environments and solid–liquid interactions are enhanced, resulting in a slight decrease in hydrophobicity. The results of this study not only provide new ideas for the low-cost and environmentally friendly preparation of superhydrophobic materials but also provide a solid theoretical basis and practical guidance for further optimising the material properties.

## 1. Introduction

With the continuous growth of China’s social economy, people’s demand for energy is rising, and this process is inevitably accompanied by a large number of production activities, which in turn has led to a series of environmental problems. Among them, the air quality pollution problem is particularly prominent and needs to be solved urgently [1]. Studies have shown that particulate matter is one of the most dangerous components of air pollution, and they are often attached to building facades, indoor environments, and furniture, posing a serious threat to human health. Based on this, it is particularly important to develop superhydrophobic materials, which have a wide range of applications, including self-cleaning [2,3], antifreeze [4], corrosion resistance [5], antimicrobial [6], and oil–water separation [7], among many others. The excellent self-cleaning properties and easy preparation of superhydrophobic materials have great potential in solving air quality problems. Superhydrophobic coatings can utilise their self-cleaning properties to effectively address the cleaning difficulties and health hazards caused by air pollution particles adhering to building surfaces. They remove pollutants through natural processes such as rainfall, significantly reducing maintenance costs and improving the human living environment [8,9]. Therefore, an in-depth study of the properties and applications of superhydrophobic materials has far-reaching practical significance and broad development prospects [8,9].

Superhydrophobic phenomena are ubiquitous in nature, such as the rolling of water droplets on lotus leaves, the water repellent property of duck feathers, and the cleanliness of butterfly wings, among other phenomena. It has been found that the surfaces of these hydrophobic materials in nature are commonly characterised by nano-scale roughness and low surface energy [10]. Based on this, one can prepare superhydrophobic surfaces by building rough structures on the surface of the material and reducing the surface energy of the material [11]. Although there are numerous methods for the preparation of superhydrophobic coatings, such as templating [12], vapour-phase deposition [13], spraying [14], etching [15], plasma-based methods [16] etc., methods such as vapour-phase deposition and etching have limitations in large-scale preparation, whereas spraying has obvious advantages in large-scale substrate applications due to its versatility, scalability, and the possibility of industrial automation. Liu et al. [17] used a one-step spraying method to prepare superhydrophobic materials and investigated the effect of different types of non-fluorinated modifiers on the wettability of the coatings, which is a simple and inexpensive preparation method, and the resulting samples exhibited excellent wear resistance and durability. In addition, the superhydrophobic materials fabricated by Yu et al. [18] through a simple spraying process showed excellent self-cleaning and anti-adhesion properties, as well as outstanding durability and stability under various harsh conditions, such as acid, alkali, various solvents, prolonged UV irradiation and outdoor placement. Therefore, the spraying method not only provides an efficient way for the industrial production of superhydrophobic materials but also opens up a broad prospect for their practical application.

Nanoparticles such as hydrophobic silica [19], titanium dioxide [20], graphene [21], carbon nanotubes [22], and metal–organic frameworks (MOFs) [23] have been successfully synthesised and efficiently adhered to the surface of various types of substrates by spraying techniques. Nevertheless, the synthesis process of nanomaterials is accompanied by unfavourable factors such as high cost, large consumption of chemicals, wastewater discharge problems, equipment investment, and energy consumption. In view of this, in order to alleviate the potential environmental pressure, researchers have turned to the use of natural mineral resources such as montmorillonite [24], diatomaceous earth [25], seafoam [26], and kaolinite [27] for the development of superhydrophobic materials. Razavi et al. [28] utilised the natural organic compounds, cinnamic acid (CA) and myristic acid (MA), to hydrophobicise seafoam nanoparticles. A hybrid coating of modified seafoam with perfluoroalkyl methacrylic acid copolymer was applied to aluminium, absorbent fabrics, glass and paper by dip coating method. The resulting coatings have a contact angle of more than 160° and a rolling angle of less than 3°, effectively reducing bacterial adhesion. The coatings remained superhydrophobic at 200 °C until they failed at 300 °C; after 30 sandpaper abrasions, the contact angles of the CA- and MA-modified coatings were reduced to 143 ± 4° and 143 ± 6°, respectively. Qu et al. [29] used stearic acid to hydrophobically modify kaolinite, and the resulting materials had good superhydrophobicity, with a high contact angle of up to 158 ± 1°, as well as good self-cleaning properties. Among these natural minerals, diatomaceous earth stands out for its economy and environmental friendliness, as well as its porous structure, large specific surface area, light weight, and good chemical stability. Nine et al. [30] used a ring mill to obtain diatomaceous earth in different sizes, dispersed graphene oxide with diatomaceous earth and TiO_2_ in tetrahydrofuran, and then attached it to substrates, such as glass and metal, by the spraying method on the surface. The contact angle of the material was as high as 170.2° due to the micro and nano structures on the surface, which increased the surface roughness. The mechanical stability of the material is good, and the superhydrophobicity is only lost after 15 sandpaper friction cycles at a constant pressure of 1.4 kPa. Diatomaceous earth is a material with a unique structure of micro- and nano-sized pores and concave structures on its surface, which gives it a natural advantage and potential for superhydrophobicity and self−cleaning. The micro and nano structures on the surface of diatomaceous earth enable water droplets to form a contact angle of almost 150 degrees or more on its surface, resulting in a superhydrophobic effect. Due to the superhydrophobic surface, the water droplets can carry away surface stains during rolling, achieving a self−cleaning effect. And its rough surface structure provides the material with good mechanical strength and stability. However, the surface of diatomaceous earth contains a large number of hydroxyl groups, and these hydroxyl groups are easy to interact with water molecules and reduce its hydrophobicity. So, by modifying diatomaceous earth, the number of hydroxyl groups on the surface can be reduced to improve its hydrophobicity and self-cleaning properties. In contrast, the structural characteristics of kaolin and seafoam are different from those of diatomaceous earth, and they do not have the micro- and nano-scale pore structure of diatomaceous earth, so they do not have the superhydrophobic and self-cleaning properties of diatomaceous earth. By modifying diatomaceous earth, its potential applications can be better utilised, such as in building materials, coatings, adsorbent materials and other fields.

In this work, we synthesised superhydrophobic powders (Dia/OTS) with a contact angle of 152.5° (±0.5°), using octadecyltriethoxysilane as a modifier and diatomaceous earth as a superhydrophobic surface roughness backbone. The superhydrophobic coating (Dia/OTS/EP coating) was further prepared using epoxy resin and polyamide curing agent as binders. This study focuses on the hydrophobicity and self-cleaning ability of the coating, including the adhesion of the coating on the surface of different substrates, such as glass, wood, and aluminium sheet, as well as an in-depth investigation of its mechanical and chemical stability, with the aim of providing theoretical support for the design of the superhydrophobic material and its application under different environmental conditions. The study of the adhesion, as well as mechanical and chemical stability, of superhydrophobic materials on the surface of various substrates not only helps to elucidate their mechanism of action and performance limitations, but it also provides scientific guidance for the optimal design and application of the materials.

## 2. Experimental Section

### 2.1. Materials

Diatomaceous earth (Dia), octadecyltriethoxysilane (OTS), epoxy resin (E44), and polyamide curing agent (650) were purchased from Macklin, and anhydrous ethanol and ammonia were purchased from Sinopharm.

### 2.2. Synthesis of Dia/OTS/EP Coatings

*Dia/OTS preparation:* Approximately 1 g of Dia was added to a mixture of 100 mL of ethanol and 20 mL of ammonia and dispersed by sonication for 30 min to uniformly disperse Dia in the solution. The homogeneously dispersed solution was magnetically stirred at room temperature and 1.2 mL of OTS was added to it and stirred at 500 r/min for 3 h. After completion of the reaction, the stirred product was centrifuged and the resulting precipitate was washed three times with ethanol and dried in an oven at 60 °C to obtain Dia/OTS powder.

**Figure 1:**
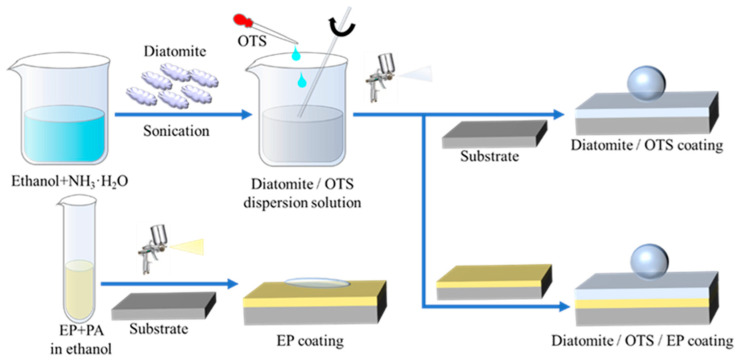
Schematic of the preparation of superhydrophobic Dia/OTS/EP coatings.

*Preparation of Dia/OTS/EP coatings:* Approximately 0.5 g of epoxy resin (EP) and 0.4 g of polyamide curing agent (PA) were dispersed in 10 mL of ethanol and sonicated for 1 h. After sonication was completed, the resulting solution was stirred for 2 h. The ethanol solution of epoxy resin and polyamide hardener was sprayed onto a clean substrate by means of an air compressor and a spray gun at a spray pressure of 0.2 MPa at a distance of 15 cm to obtain an EP coating. After curing at room temperature for 2 h, an ethanol suspension of Dia/OTS powder was sprayed onto the surface of the EP coating and cured at room temperature for 24 h to obtain the Dia/OTS/EP coating. The experimental preparation process is shown in Figure 1.

### 2.3. Characterisation

*Morphology and structure:* In this experiment, the surface morphology of the samples was observed using a scanning electron microscope (SEM, LMS, Praha, Czech Republic). The crystal structure of the materials was determined using an X-ray diffractometer (XRD, SmartLab 9 kW, Tokyo, Japan). A Fourier-Transform Infrared Spectrometer (FTIR, Nicolet iS20, Waltham, MA, USA) was used to test, analyse, and identify the chemical composition and functional groups in the samples. An X-ray photoelectron spectrometer (XPS, ESCALAB 250Xi, Waltham, MA, USA) was used to characterise the elemental composition of the material surfaces. The water contact angle of the sample surface was measured using a contact angle meter (CA, OCA15pro, Berlin, Germany).

*Adhesion test:* We used the spraying method to develop superhydrophobic materials, with diatomite as the skeleton and OTS as the modifier, characterised their physical phase composition, microscopic morphology, and surface chemical state, and tested their hydrophobicity on the surfaces of various. First, we used a nail rake to draw vertical grid lines on the coating surface. Then, we closely affixed tape along one direction of the grid. After 5 min, we peeled off the tape at a 60° angle and used a brush to remove any residue. Finally, we evaluated the adhesion grade based on the assessment table provided with the rake and the degree of coating peeling.

*Mechanical Stability Test:* The mechanical stability properties of the Dia/OTS/EP coatings were measured by means of a sandpaper abrasion test and a scribing tape test. The sandpaper (1000 grit) abrasion test consists of placing a glass sheet coated with Dia/OTS/EP face down on sandpaper so that the surface of the coating is in contact with the surface of the sandpaper, and then placing a 200 g weight on top of it and rubbing the coating and the weight as a unit back and forth for 10 cm at a speed of 1 cm/s. One cycle is recorded as one cycle, and each cycle is measured as one measurement of the contact angle of the coating. One round trip is recorded as one cycle, and the contact angle of the coating is measured once per cycle. The tape test is performed by applying 3M^TM^ #600 tape to the coated surface, removing the tape after 5 min, and measuring the contact angle of the coating after sweeping the coated surface with a brush, with one cycle for each ‘adhesion-peel’ process. See Figure S6 for a schematic diagram.

*Chemical Stability Testing:* The coatings were placed in 1 M hydrochloric acid, pure water, and 1 M sodium hydroxide solution, respectively, and taken out after 24 h. The contact angle sizes were measured and compared with those of the original coatings to determine the chemical stability of the coatings under extreme acid and alkali environments.

*Self-cleaning test:* In order to investigate the self-cleaning properties of the coatings prepared in the experiments, water-soluble methyl blue and non-water-soluble iron powder were used as contaminants placed on the surface of the coatings, respectively. Water drops were then dropped on the inclined coating surface at an angle of about 10° to remove the contaminants from the surface. The self-cleaning performance was judged based on the degree of cleaning.

*Molecular dynamics simulation flow:* The CVFF force field is used, the initial velocity is Random, the time step of the computational process is 1 fs, the truncation radius is set to 12.5 Å, and the total duration of the simulation process is 1000 ps. The NVT system is used to control the temperature of the system by the Nose–Hoover method, and the trajectory information is output every 2000 steps, and all the models are analysed to analyse the trajectory of the last 100 ps output and the thermodynamic properties. (Modelling [31,32,33,34,35] and the corresponding results Figures S1–S3 and Table S1 are detailed in the Supplementary Information.)

## 3. Results and Discussion

### 3.1. Microstructural and Morphological Analysis of Dia and Dia/OTS

Based on the simulation results (see Supplementary Information for detailed results), the hydrophobicity of diatomaceous earth modified by OTS is significantly improved. Therefore, we used the spraying method to develop superhydrophobic materials with diatomite as the skeleton and OTS as the modifier, characterised their physical phase composition, microscopic morphology, and surface chemical state, and tested their hydrophobicity on the surfaces of various substrates.

#### 3.1.1. Dia and Dia/OTS Surface Morphology Analysis

**Figure 2:**
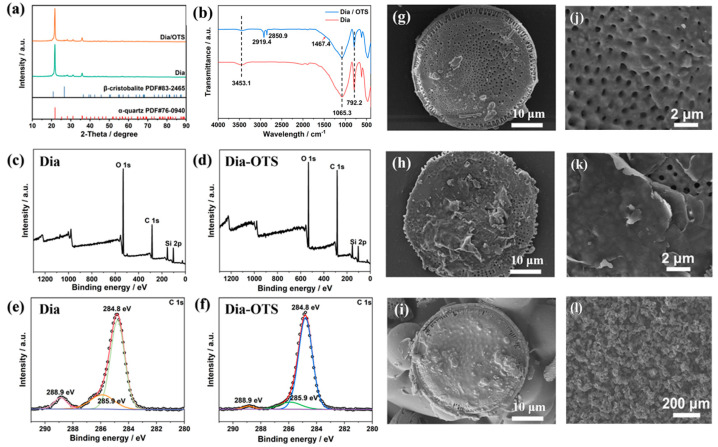
(**a**) XRD diagrams of Dia and Dia/OTS powders; (**b**) FTIR diagrams of Dia and Dia/OTS; (**c**,**d**) XPS full spectra of Dia and Dia-OTS; (**e**,**f**) XPS C 1s spectra of Dia and Dia-OTS; (**g**,**j**) scanning electron micrographs of Dia; (**h**,**k**) scanning electron micrographs of Dia-OTS; (**i**,**l**) scanning electron micrographs of Dia/OTS/EP coatings.

Figure 2g,h,j,k shows the SEM images of Dia and Dia/OTS, aiming to analyse the effect of OTS treatment on the surface structure of diatomite. From Figure 2g,j, it can be observed that the untreated diatomite has a typical porous disc-shaped structure with a diameter distribution between 15 and 40 µm and a pore size of about 500–800 nm. The surface of these disc-shaped diatomite is smooth, and the pores are well defined and tightly arranged. After the OTS modification treatment, the basic morphology of the diatomite remained unchanged, but the surface characteristics changed significantly: the coverage of the OTS layer made the surface of the diatomite rougher, and this phenomenon confirmed that the polysiloxane successfully modified the surface of the diatomite.

#### 3.1.2. Physical Phase Analysis of Dia and Dia/OTS Powders

The X-ray diffraction (XRD) patterns of diatomite and its modified product Dia/OTS are shown in Figure 2a. The analysis of the maps shows that the main component of diatomite is silicon dioxide (SiO_2_), specifically α-quartz, β-cristobalite, and an indeterminate SiO_2_ phase. For the modified diatomaceous earth, the crystal structure did not change significantly, which indicates that the modification process of octadecyltriethoxysilane did not affect the crystal structure of diatomaceous earth. This is consistent with the SEM results. In the XRD spectra, the diffraction peaks located at 2θ = 21.8°, 28.2°, 31.1°, and 35.9° correspond to the (101), (111), (102), and (200) crystal planes of β-cristobalite, respectively, whereas the diffraction peaks located at 2θ = 20.9° and 26.6° are attributed to the (100) and (101) crystal planes of α-quartz, respectively [36]. The XRD pattern shows a distinct diffraction peak at 2θ = 20°, indicating the presence of an amorphous phase in the material; additionally, the diffraction peak at 2θ = 43° is attributed to silica [37].

#### 3.1.3. Surface Chemical State Analysis of Dia and Dia/OTS Materials

The results of Fourier-Transform Infrared Spectroscopy (FTIR) and X-ray Photoelectron Spectroscopy (XPS) analyses together reveal the successful preparation of Dia/OTS materials. The FTIR analysis revealed that the modification of the diatomaceous earth surface resulted in significant changes in the functional groups. Specifically, the intensity of the -OH stretching vibrational peak, which was originally significant at 3453.1 cm^−1^, has decreased, suggesting that the number of hydroxyl groups on the surface has been reduced. In addition, the asymmetric stretching vibrational peak of the Si-O-Si bond (1065.3 cm^−1^) and the bending vibrational peak of -OH (792.2 cm^−1^) also showed a tendency to weaken, which reflected the reorganisation of the surface structure of diatomite. In the infrared spectra of Dia/OTS materials, we also found new absorption peaks at 2919.4 cm^−1^ and 2850.9 cm^−1^, which are related to the stretching vibration of the -CH bond, while the peak at 1467.4 cm^−1^ is related to the bending vibration of -CH_2_. These newly appeared characteristic peaks indicated that the hydroxyl groups on the surface of diatomaceous earth underwent a condensation reaction with octadecyltriethoxysilane, resulting in the formation of a layer of polysiloxane on the surface of diatomaceous earth.

Figure 2c–f shows the XPS results for Dia and Dia/OTS materials, while Table 1 demonstrates the elemental composition and content. In Dia/OTS, the intensity of the C 1s peak increases and the carbon content rises from 40.43% to 61.81%, while the oxygen content decreases from 41.20% to 25.26%, and the silicon content also decreases from 18.37% to 12.93%, which suggests that the chemical constituent elements of octadecyltriethoxysilane (OTS), C, H, O, and Si, have been successfully introduced into the Dia/OTS material. Table 2 presents the electron-bond energies for each functional group. The fine scanning of the C 1s peaks were finely scanned and showed that the CH_2_-CO and O-C=O peaks were weakened and the C-C/C-H peaks were enhanced in Dia/OTS, indicating that polysiloxane was grafted to diatomaceous earth through the reaction of OTS with hydroxyl groups. The XPS results were in agreement with the FTIR analyses, which confirmed the successful modification of the Dia/OTS material. Furthermore, the calculation results indicate that the covalent modification of the silica surface by octadecyltrichlorosilane (OTS) leads to a significant increase in the total energy of the system in an aqueous environment, approximately 8802 kcal/mol. This substantial energy increase stems from the strong hydrophobic effect triggered by the introduction of hydrophobic alkyl chains, theoretically confirming that OTS modification is the key driving force behind the transformation of the surface from hydrophilic to hydrophobic.

### 3.2. Application Performance Study of Dia/OTS/EP Materials

The hydrophobicity of the OTS-modified diatomaceous earth is significantly improved. The presence of CH_3_-(CH_2_)_17_-Si (OH)_2_-groups reduces the number of exposed hydroxyl groups on the SiO_2_ surface and effectively inhibits the formation of hydrogen bonding between the water molecules and the hydroxyl groups on the surface, which leads to a more loose distribution of water molecules on the SiO_2_-OTS surface and a longer diffusion distance. In addition, the introduction of CH_3_-(CH_2_)_17_-Si (OH)_2_-groups reduces the negative electronegativity of the surface, weakens the electrostatic interactions with water molecules, and thus reduces the strength of solid–liquid interactions in the system, which leads to the weakening of the adsorption of water molecules on the surface of the SiO_2_-OTS, and thus further strengthens the hydrophobicity of the SiO_2_-OTS (see Supplementary Information for details on the simulation process). In order to verify this idea, we prepared a superhydrophobic coating using diatomaceous earth as the skeleton, OTS as the modifier, and epoxy resin as the adhesion agent by the spraying method. The aim was to investigate the application properties of the coating, specifically hydrophobicity, adhesion, self-cleaning, mechanical stability, and chemical stability.

#### 3.2.1. Hydrophobicity Study of Dia/OTS/EP Materials

**Figure 3:**
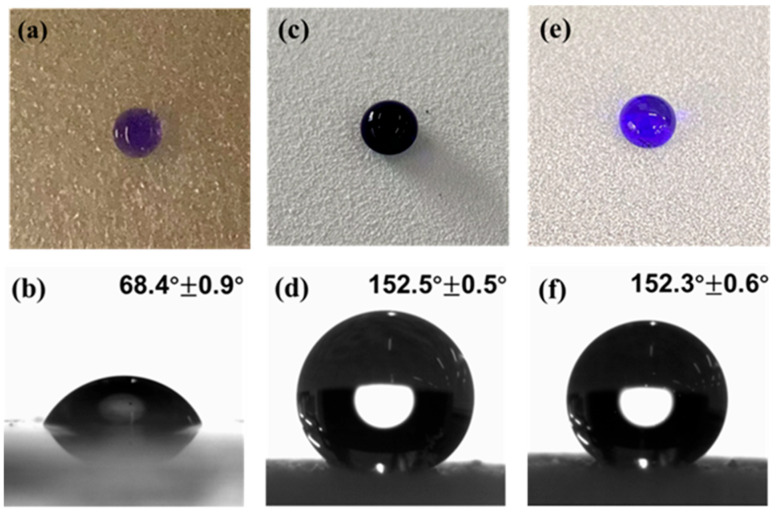
Physical diagrams of methyl blue stained water droplets in (**a**,**b**) EP/PA coating, (**c**,**d**) Dia/OTS coating, and (**e**,**f**) Dia/OTS/EP coating with contact angles.

Figure 3 shows the physical photographs and contact angle measurements of water droplets on the surface of EP/PA coating, Dia/OTS coating, and Dia/OTS/EP coating. It can be observed that the cured epoxy coating has a contact angle of less than 90° with the water droplet, which shows hydrophilic characteristics. At the same time, both Dia/OTS and Dia/OTS/EP coatings show excellent superhydrophobicity with droplet contact angles exceeding 150°. A photograph of the material in water can be found in Figure S4.

**Figure 4:**
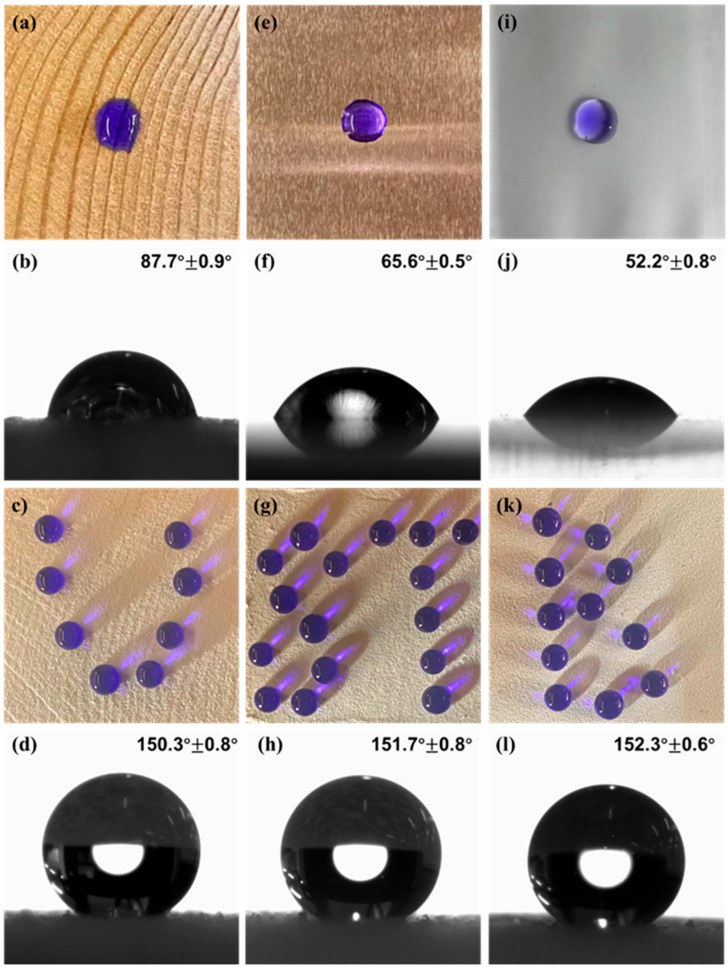
Physical views and droplet contact angles of (**a**,**b**) wood, (**e**,**f**) aluminium, (**i**,**j**) and glass surfaces; physical views and droplet contact angles of Dia/OTS/EP coatings attached to (**c**,**d**) wood, (**g**,**h**) aluminium, (**k**,**l**) and glazing surfaces.

Figure 4 clearly shows the surface properties of common materials in our daily life. It can be observed that the surface contact angles of water droplets with wood, aluminium, and glass are 87.7 ± 0.9°, 65.6 ± 0.5°, and 52.2 ± 0.8°, respectively, which are all less than 90°, and accordingly it can be judged that these materials are hydrophilic materials. On the surface of these substrates, the droplets showed a more regular hemispherical shape. After removing the methyl blue solution, a slight residue of methyl blue dye was left on the surface of each substrate. In particular, the wood surface, with its characteristic streak-like depressions, allowed the droplets to be gradually absorbed by the wood over time after contacting the wood surface. Further analysing Figure 4, the Dia/OTS/EP-coated surfaces of wood, aluminium, and glass treated with static contact angles of 150.3 ± 0.8°, 151.7 ± 0.8° and 152.3 ± 0.6°, respectively, showed superhydrophobicity. Water droplets rolled off quickly on these surfaces with no dye residue. The superhydrophobicity of the coatings was attributed to high roughness and low surface energy. Differences in hydrophobicity between substrates may be due to different surface structures and properties. For example, wood surface streaks lead to a Wenzel state in parts of the Dia/OTS/EP coating, resulting in a slightly lower contact angle. The experimental results confirm that Dia/OTS/EP coatings can be effectively imparted superhydrophobicity to common substrates by applying them to the surface in a two-step spraying method.

#### 3.2.2. Dia/OTS/EP Coating Adhesion Study

**Figure 5:**
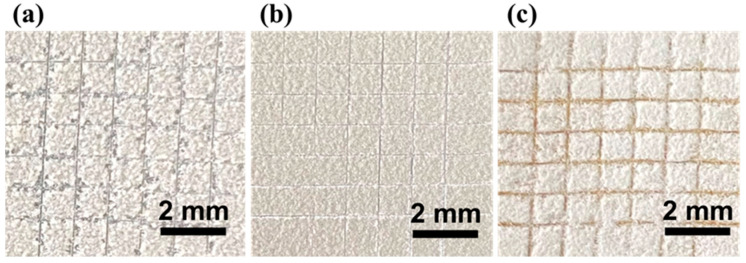
Dia/OTS/EP coating adhesion test charts on various substrate surfaces: (**a**) glass; (**b**) aluminium sheet; (**c**) wood.

Figure 5 illustrates the results of the adhesion tests of Dia/OTS/EP coatings on three different substrate surfaces: glass, wood, and aluminium. Combined with the data analysis in Table 3, we can observe that the coating applied to the glass surface showed partial peeling at the cut intersections, and its adhesion was rated as Level 2, while the coatings applied to the aluminium and wood surfaces showed only slight peeling at the cut intersections, and their adhesion was rated as Level 1. Overall, Dia/OTS/EP coatings demonstrated good adhesion on all test substrates.

Epoxy resins are known for their high crosslink density and degree of cure, excellent corrosion resistance, and high modulus of elasticity [36]. The amine hydrogen in the polyamide reacts with the epoxy group to form a strong chemical bond. Upon addition of the polyamide curing agent, the epoxy resin forms a highly crosslinked, three-dimensional network by nucleophilic addition reaction, which is firmly embedded in the Dia/OTS powder. Epoxy resins contain a variety of polar groups and polyamides contain fatty acid carbon chains and amino groups, which give cured epoxy resins excellent adhesion. Glass, whose main component is silica, has hydroxyl groups on its surface, with which polyamide−cured epoxy resin reacts easily to achieve good adhesion [40]. Aluminium plate has an Al_2_O_3_ layer and hydroxyl groups on the surface, and wood contains a variety of reactive functional groups, all of which can form a strong chemical bond with the epoxy resin to enhance adhesion. The epoxy resin adhesion is related to the surface roughness of the substrate, and the glass surface is smoother, which may be the reason for the weaker adhesion of the coating on the glass.

#### 3.2.3. Study of Self-Cleaning Properties of Dia/OTS/EP Coatings

**Figure 6:**
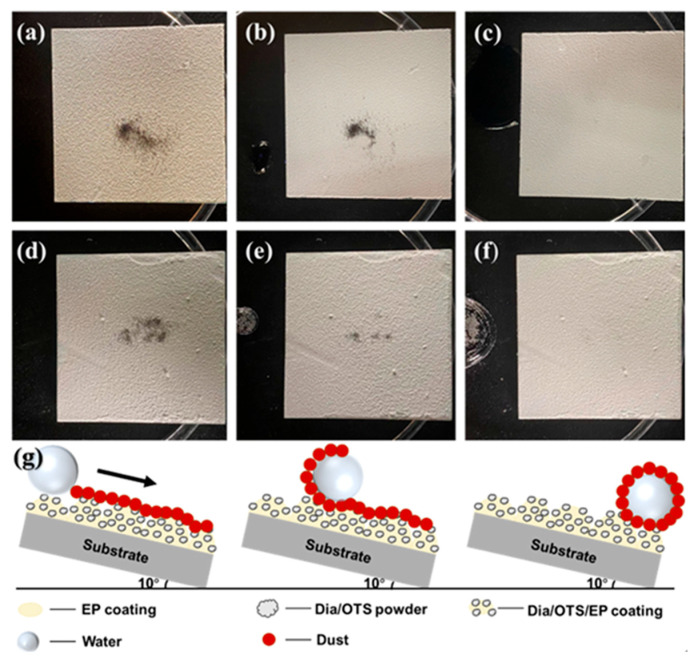
(**a**–**c**) Test procedure using methyl blue as a contaminant: (**a**) coating state before adding water droplets, (**b**) coating state after adding one water droplet, (**c**) coating state after adding multiple water droplets; (**d**–**f**) Test procedure using iron powder as a contaminant: (**d**) coating state before adding water droplets, (**e**) coating state after adding one water droplet, (**f**) coating state after adding multiple water droplets; (**g**) Schematic diagram of the self-cleaning process.

The results of the self-cleaning test are shown in Figure 6. As the water droplets rolled off, both the water-soluble methyl blue and the water-insoluble iron powder were effectively removed from the coating surface. Since methyl blue is soluble in water, when a water droplet touches the surface of the Dia/OTS/EP coating and rolls to the contaminant, the methyl blue quickly dissolves in the droplet and is carried away from the coating as the droplet moves. As the number of water droplets increased, the water-soluble contaminant was completely removed from the coating surface, and the surface no longer showed the blue colouring of methyl blue.

Figure 6g shows a schematic of the self-cleaning process of the Dia/OTS/EP coating. The surface of the coating is dotted with numerous protrusions and pores, and these microstructures are capable of trapping air and forming an air layer in the solid–liquid phase, which reduces the adhesion of water droplets on the surface of the coating. As a result, when the coating is slightly tilted, the water droplets are able to roll off easily [41]. On the other hand, even though iron powder is insoluble in water, it can be effectively carried away from the coating surface during the rolling of water droplets. This is because the adhesion of the contaminant to the water exceeds its adhesion to the coating, and therefore the contaminant is removed as the water droplet rolls over the coating. From this, we can conclude that Dia/OTS/EP coatings have excellent self-cleaning properties and are able to effectively remove contaminants from surfaces and can be used on a wide range of real-life surfaces for self-cleaning and dust removal.

#### 3.2.4. Dia/OTS/EP Coating Application Stability Study

**Figure 7:**
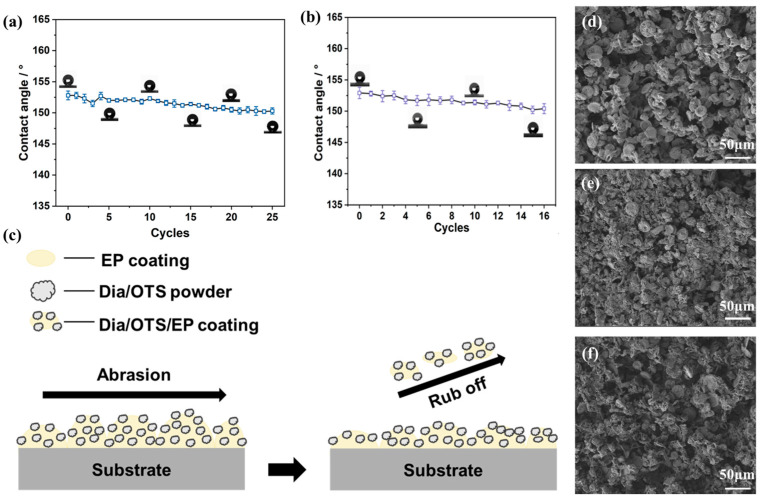
(**a**) Change in contact angle of Dia/OTS/EP coated surfaces for 25 cycles of sandpaper friction; (**b**) Change in contact angle of Dia/OTS/EP coated surfaces for 16 cycles of ‘adhesion-peel’; (**c**) Schematic representation of Dia/OTS/EP coated surfaces before and after abrasion; (**d**) Scanning electron microscope image of Dia/OTS/EP coating before wear; (**e**) Scanning electron microscope image of Dia/OTS/EP coating after 25 cycles of sandpaper friction; (**f**) Scanning electron microscope image of Dia/OTS/EP coating after 16 cycles of ‘adherence-peeling’.

Figure 7a reveals the static contact angle variation in the Dia/OTS/EP coating as it undergoes wear. The contact angle gradually decreases as the number of abrasion cycles increases, but only slightly decreases from 152.8 ± 0.1° to 152.0 ± 0.3° in the first ten cycles and then remains stable. It remained stable until the 25th cycle at 150.3 ± 0.2°, showing that the coating maintained its superhydrophobicity. This result highlights the excellent abrasion resistance of the Dia/OTS/EP coating, which is attributed to the strong cohesion of the epoxy resin and the high abrasion resistance of the diatomaceous earth. Figure 7b shows the change in contact angle of the Dia/OTS/EP coating after 16 cycles of ‘adhesion-peel’. After the full 16 cycles, the contact angle stabilised at 150.2 ± 0.2°, still exhibiting superhydrophobic properties. After the abrasion test, the water droplets remained in a spherical shape on the surface of the coating, which further proves the excellent mechanical properties and strong adhesion of the Dia/OTS/EP coating. Figure 7c demonstrates the surface state of the Dia/OTS/EP coating before and after abrasion. When prepared, Dia/OTS powder was sprayed as an ethanol suspension on the incompletely cured EP coating, and part of the powder was embedded in the coating and part of it adhered to the surface. Upon abrasion or stripping, the surface Dia/OTS powder and part of the epoxy resin may come off. However, the embedded powder keeps the surface rough and maintains superhydrophobicity. Upon abrasion, the hydrophilic EP coating is exposed, resulting in a decrease in contact angle.

Figure 7d–f shows that the Dia/OTS/EP coating maintains micrometre roughness after wear, but the number of protrusions and pores decreases and the diatomite particles break down. Under increased abrasion, sandpaper breaks down the surface structure, decreasing the number of protrusions and pores, as well as the contact angle. Hydrophilic epoxy is exposed, weakening hydrophobicity. After adhesion–exfoliation damage, the surface micromorphology changes little, maintaining micrometre-scale roughness and decreasing the number of protrusions. Figure 7f shows that the pore area increases and surface roughness decreases, which is due to the shedding of Dia/OTS powder, resulting in reduced air capture and hydrophobicity. Partial shedding exposes the hydrophilic epoxy and reduces the contact angle. Nevertheless, the coating maintains roughness without structural collapse and still exhibits superhydrophobic properties.

**Figure 8:**
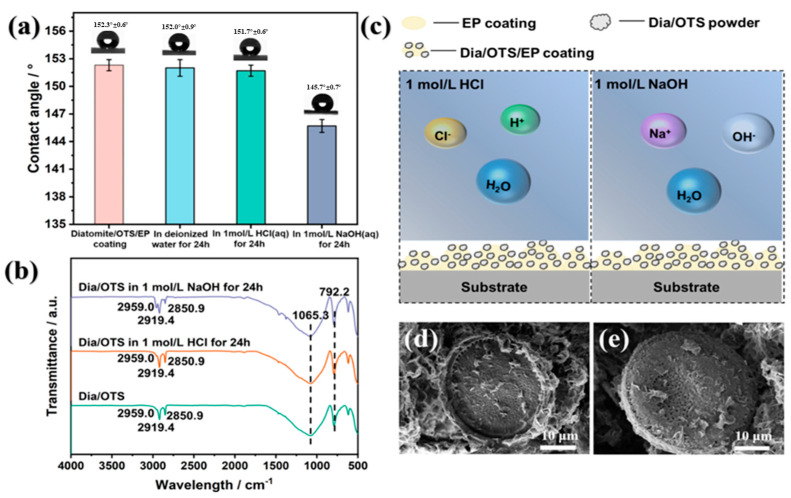
(**a**) Wettability changes in Dia/OTS/EP coatings in different chemical states; (**b**) Dia/OTS and IR spectra of Dia/OTS after immersion in acid–base solution for 24 h; (**c**) Schematic diagram of the mechanism by which Dia/OTS/EP coatings remain hydrophobic in acid–base solution; (**d**) Scanning electron micrographs of Dia/OTS/EP coatings after immersion in 1 M HCl solution for 24 h; (**e**) Scanning electron micrographs of Dia/OTS/EP coatings after immersion in 1 M NaOH solution.

Figure 8a demonstrates the stability of Dia/OTS/EP coatings in different chemical environments. After 12 h of immersion in deionised water, the contact angle of the coatings remained largely unchanged, showing stable hydrophobicity. After 24 h of immersion in 1 M hydrochloric acid, the contact angle slightly decreased from 152.3 ± 0.6° to 151.7 ± 0.2°, but the coating still maintained its superhydrophobic properties. In contrast, the contact angle decreased from 152.3 ± 0.6° to 145.7 ± 0.1° after 24 h of immersion in 1 M sodium hydroxide solution. In addition, the coating maintained a high contact angle when exposed to the natural environment for a long period of time, demonstrating its excellent durability. The decrease in hydrophobicity of the coatings under acidic and alkaline conditions was mainly due to the hydrolysis of polysiloxanes and the destruction of Si-O bonds, which was especially significant under alkaline conditions. The silica component of diatomaceous earth is susceptible to erosion in sodium hydroxide, disrupting the coating structure and reducing the contact angle. This is because the ions present in the electrolyte solution effectively disrupt the highly ordered water molecule structure near the hydrophobic interface. Na^+^ in NaOH solution and Cl^−^ in HCl solution migrate to the hydrophobic interface, disrupting the hydrogen bond network, increasing the entropy in the interface region, and thereby significantly reducing the system’s free energy. Additionally, under alkaline conditions, the SiO_2_ substrate surface may carry more negative charges (Si-O^−^), further attracting Na^+^ ions to form a hydrophilic double layer, resulting in a smaller contact angle in alkaline solutions (see Supplementary Information Figure S5 and Table S2 for simulation details.)

Figure 8b demonstrates the infrared spectra of Dia/OTS coatings and their immersion in acid–base solutions for 24 h. Characteristic peaks associated with -CH bonds (2959.0 cm^−1^, 2919.4 cm^−1^ and 2850.9 cm^−1^) and absorption peaks of Si-O-Si and -OH groups (1065.3 cm^−1^ and 792.2 cm^−1^) appeared in the spectra. The enhanced intensity of the Si-O-Si and -OH peaks after acid–base immersion may be due to the hydrolysis of polysiloxanes, the breaking of Si-O bonds, and the increase in the number of hydroxyl groups on the surface, which led to the decrease in the contact angle. Figure 8d,e demonstrates the SEM photographs of Dia/OTS/EP coatings after immersion in 1 M HCl solution and 1 M NaOH solution for 24 h. It can be observed that the overall morphology of the coatings was not damaged despite the strong acid and alkali immersion, and the surfaces still retained the protrusions and pores, maintaining the micron-level roughness, and the coatings were still covered with polysiloxanes, which maintained the characteristics of low surface energy and high roughness, and thus the coatings still exhibited excellent hydrophobicity after acid and alkali immersion. Figure 8c shows that the low surface energy and high roughness of the Dia/OTS/EP coating form an air cushion that hinders the contact of corrosive liquids, reduces the penetration of OH^−^ and H^+^ particles, and lowers the damage rate. The chemical stability of diatomaceous earth maintains structural stability in acidic environments, and polyamide curing enhances the chemical inertness of the epoxy resin, conferring excellent corrosion and water resistance to the coating.

## 4. Conclusions

In summary, we have successfully developed a low-cost superhydrophobic coating using diatomaceous earth (Dia) as the backbone, octadecyltrichlorosilane (OTS) as the modifier, and epoxy resin (EP) as the binder by means of a facile spraying technique. The results showed that Dia/OTS powder was effectively adsorbed on the substrate surface by the epoxy resin, forming a stable micrometre-scale rough structure, which made the coating contact angle as high as 152.3 ± 0.6°. The coating adheres firmly to a wide range of surfaces, including glass, wood, and aluminium, giving them superhydrophobic properties. Even after 25 sandpaper abrasion cycles or 16 tape stripping cycles, the coating maintains its surface roughness and contact angle of over 150°. In terms of chemical stability, the diatomaceous earth-based superhydrophobic coatings showed good hydrophobicity after 24 h of acid and alkaline corrosion, with contact angles of 151.7 ± 0.2° and 145.7 ± 0.1°, respectively. In addition, the coating demonstrated excellent self-cleaning capabilities, effectively removing surface contaminants. The excellent performance of the superhydrophobic coatings provides an economical and practical way for the preparation and application of superhydrophobic coatings, which is expected to promote the development of related fields and open up new paths for solving practical problems.

## Figures and Tables

**Table 1:** Dia and Dia/OTS surface elemental composition and percentage content.

Atomic %	C	O	Si
Dia	40.43	41.20	18.37
Dia/OTS	61.81	25.26	12.93

**Table 2:** Electron binding energy of different groups.

Base Group	Combined Energy [38,39]
C-C/C-H	284.8 eV
CH2-CO	285.9 eV
O-C=O	288.9 eV

**Table 3:** Hundred-gram method test results.

Substrates	Adhesion Rating
glass	Level 2 (less than 15 per cent shedding)
aluminium sheet	Level 1 (less than 5 per cent shedding)
wood	Level 1 (less than 5 per cent shedding)

## Data Availability

The original contributions presented in this study are included in the article/Supplementary Material. Further inquiries can be directed to the corresponding authors.

## Supplementary Materials

The following supporting information can be downloaded at: https://www.mdpi.com/article/10.3390/ma18174209/s1, Figure S1: (a) OTS hydrolysis products; (b) SiO_2_-OH model; (c) SiO_2_-OTS model; (d) SiO_2_-OH surface in water; (e) SiO_2_-OTS surface in water; Figure S2: Relationship between OTS dosage and contact angle; Figure S3: (a) Radial distribution functions of H and O atoms in SiO_2_-OH and SiO_2_-OTS systems; (b) iInteractions with water, van der Waals effect, and total energy of the system at the SiO_2_-OH and SiO2-OTS surfaces; (c) Z-direction distributions of water molecules at the SiO_2_-OH and SiO_2_-OTS surfaces; (d) equilibrium configuration on the SiO_2_-OH surface; (e) equilibrium configuration on the SiO_2_-OTS surface; (f) equilibrium configuration of water on the SiO_2_-OH surface; (g) equilibrium configuration of water on the SiO_2_-OHS surface; Figure S4: (a) Dispersion test of Dia in water; (b) Dispersion test of Dia/OTS powder in water; (c) Physical image of Dia/OTS/EP coating before immersion in water; (d) Physical image of Dia/OTS/EP coating immersed in water; Figure S5: (a) Number of water molecules along the *z*-axis direction on the SiO_2_-OTS surface in 1 mol/L HCl solution; (b) Distribution of water molecules along the *z*-axis direction on the SiO_2_-OTS surface in 1 mol/L NaOH solution; (c) Mean-square displacements (MSDs) on the SiO_2_-OH surface; (d) MSDs on the SiO_2_-OTS surface; (e) Pure water in the water molecules on the SiO_2_-OH surface and the SiO_2_-OTS surface; (f) MSD of water molecules in 1 mol/L HCl solution on the SiO2-OH surface and the SiO_2_-OTS surface; (g) MSD of water molecules in 1 mol/L NaOH solution on the SiO_2_-OH surface and the SiO_2_-OTS surface; (h) MSD of different solutions with the SiO_2_-OTS solid–liquid interactions, van der Waals, and total energy of the system; Figure S6: Schematic of sandpaper abrasion experiment. Table S1: Atom types in the CVFF; Table S2: diffusion coefficients of water molecules in different systems (cm^−5^/s).
