# Supplementary material for: Experimental and Simulation Investigation of Octadecyltriethoxysilane-Decorated Diatomaceous Earth Coatings with Enhanced Superhydrophobic and Self-Cleaning Properties
Source: Materials (Basel). 2025 Sep 8;18(17):4209. doi: 10.3390/ma18174209 (PMC12430288; doi:10.3390/ma18174209)
Supplement: Supplementary file 1 [file materials-18-04209-s001.zip › materials-3805747-supplementary.pdf]

Supplementary Information for

Experimental and Simulation Investigation of Octadecyltriethoxysilane-Decorated Diatomaceous Earth Coatings with Enhanced Superhydrophobic and Self-Cleaning Properties

Aijia Zhang [1,2], Nan Xiao [1,2], Kunjie Yuan [1,2,*] and Wenbin Cao [1,3,*]

[1] School of Materials Science and Engineering, University of Science and Technology Beijing, Beijing 100083, China
[2] School of Energy and Environmental Engineering, Hebei University of Technology, Tianjin 300401, China
[3] Tianjin College, University of Science and Technology Beijing, Tianjin 301830, China
* Correspondence: kyuan@hebut.edu.cn (K.Y.); wbcao@ustb.edu.cn (W.C.)

Molecular dynamics calculations

Selection of force fields

The empirical expression of the potential energy function of the force field has an important influence on the reliability of the calculation results, since the chosen force field is closely related to it. The Consistent Valence Force Field (CVFF) force field is chosen for the calculations in this paper. The detailed form of the CVFF force field is as follows [1]: $U = \sum_{bond} K_b(b - b_0)^2 + \sum_{angle} K_\theta(\theta - \theta_0)^2 + \sum_{torsion} K_\phi[1 + \cos(n\phi - \phi_0)] + \sum_{out\text{-}of\text{-}plame} K_\chi[1 + \cos(n\chi - \chi_0)] + \sum_{L\text{-}J}\left[\left(\frac{A_{ij}}{r_{ij}}\right)^{12} - \left(\frac{B_{ij}}{r_{ij}}\right)^{6}\right] + \sum_{coulomb}\frac{q_i q_j}{\varepsilon_0 r_{ij}}$, where K_b, K_θ, K_φ, and K_χ are force constants; b_0, θ_0, φ_0, and χ_0 are equilibrium bond lengths, bond angles, torsion angles, and out-of-plane angles; b, θ, φ, and χ are bond lengths, bond angles, torsion angles, and out-of-plane angles, respectively; n is a periodicity parameter; and A_{ij} and B_{ij} are the square root of the product of A_i and A_j and B_i and B_j. ε_0 is the effective permittivity; r_{ij} is the distance between the atoms with a charge of q_i and those with a charge of q_j.

Selection of Ligatures

A class of systems that have the same structure and properties and have a certain state of motion and are independent of each other is known as a system of systems. In molecular dynamics, various types of systems can be dealt with, and in this experiment, the NVT system is used, where N denotes the number of particles, V denotes the volume, and T denotes the temperature, i.e., the number of particles, the volume, and the temperature of the system are kept constant.

Selection of numerical solution of Newton's equations

By resolving the equations of motion of the system, the complete trajectory of the position and velocity of each particle in the system over time can be obtained, and the Verlet algorithm is used in this experiment to resolve the equations of motion.

Selection of periodic boundary conditions

In the calculation process, in order to keep the density of the system unchanged, the periodic boundary conditions are usually used to ensure that the density in the calculation system is consistent with the density in the experimental observation. In this experiment, the periodic boundary conditions are chosen as ppp, i.e., the three directions x-y-z in the simulation box are all periodic boundaries.

Molecular dynamics simulation flow

To carry out molecular dynamics simulations, it is first necessary to create a suitable model and construct a simulation box of the right size according to the properties of the object under study. After determining a suitable system, the temperature, starting velocity, and simulation time of the system must be set. During the simulation of the system, the velocity and position of the particles in the system change with time, which results in the energy within the system always being in a constant state of flux. For the calculation results of different moments, it can be determined what kind of thermal equilibrium state the whole system is in. When the value of the energy change of the whole system fluctuates within 10%, the system is considered to be in thermal equilibrium. If the system is in non-equilibrium or unstable state, the distribution of physical quantities within the system can be

controlled by changing the initial conditions. When the system reaches thermodynamic equilibrium, the trajectories of particles in the system and other thermodynamic properties can be statistically processed, so as to realise the analysis of the macroscopic properties of the research object. The detailed process is described below:

First of all, the research objective is to establish the corresponding model, set its initial conditions and calculation parameters (system temperature, initial speed, force field, simulation time, etc.), the combined force and acceleration of the particles are calculated so as to obtain the next moment of the particle's speed and position before finally outputting the results of the calculations, and when the time of the calculation reaches the set time, the calculations are finished. In order to avoid the emergence of errors due to repeated calculation of particle forces, the non-bonded remote force calculation must use the truncated radius method. This method stems from the most mirror image idea that if the distance between two molecules is greater than a specific truncation radius, their interaction will not be accounted for. In practical applications of molecular dynamics simulations, the method is of great theoretical and practical importance. Typically, atoms need to be careful in determining the truncation radius, the value of which should not be more than half of the box length; the usually chosen truncation radius is around 10-15 Å. The truncation radius of a molecule should not be more than half of the box length.

The computational parameters used in this experiment are as follows: the CVFF force field is used, the initial velocity is Random, the time step of the computational

process is 1 fs, the truncation radius is set to 12.5 Å, the total duration of the simulation process is 1000 ps, the NVT system is used, the temperature of the control system is controlled by the Nose–Hoover method, the trajectory information is outputted once in every 2,000 steps, and the analyses of all models are performed. Trajectory and thermodynamic properties of the last 100 ps are outputted.

Modelling

A model of diatomaceous earth surface with different surface functional groups and a solid–liquid interface model of this surface with water molecules were successfully constructed using Materials Studio software. Subsequently, the constructed model was simulated in detail using LAMMPS software. After the calculation, the trajectory files generated during the simulation process were professionally processed using Ovito software.

Comparison of diatomite models before and after modification

Given that the main component of diatomaceous earth is SiO_2 and its surface is rich in hydroxyl groups, the SiO_2 model with hydroxyl groups on the surface was selected as a representative model of diatomaceous earth in this study. In the modification study, we selected octadecyltriethoxysilane (OTS) as the modifier. The SiO_2 model was constructed with reference to the research results of Yaphary Y L [2]. In this study, considering that the long carbon chains in OTS can be stably and firmly bonded to hydroxylated surfaces, all silane branches in the OTS molecule were replaced by -OH groups by hydrolysis during the model construction process. On this basis, we grafted the CH_3-$(CH_2)_{17}$-Si-$(OH)_3$ [3] structure on the diatomaceous earth

model, thus obtaining the modified octadecyltriethoxysilane diatomaceous earth model.

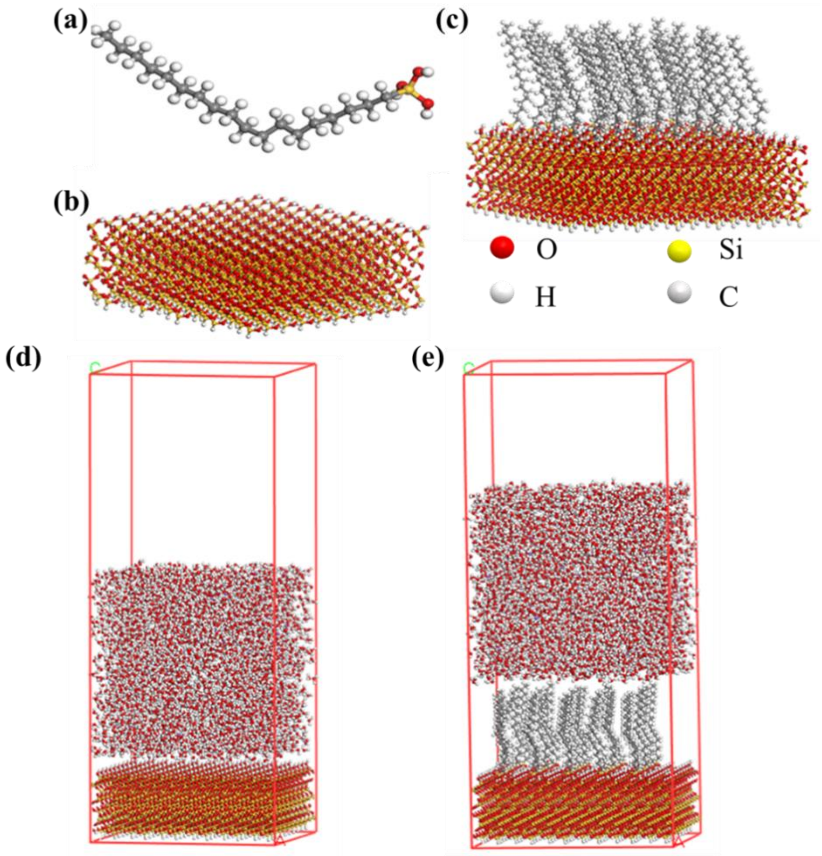

Figure S1. (a) OTS hydrolysis products; (b) SiO_2-OH model; (c) SiO_2-OTS model; (d) SiO_2-OH surface in water; (e) SiO_2-OTS surface in water.

The process of building the diatomite model before and after modification is shown below: SiO_2 with α-quartz structure was imported in Materials Studio with lattice parameters of a=b=4.916 Å, c=5.405 Å, and α=β=γ=90°. The (001) crystal surface was cut and constructed with dimensions of 49.13×49.×16.22 $Å^3$ and hydrogen atoms were added to the oxygen atoms on the surface to make hydroxyl groups present on the surface of the silica as shown in **Figure S1(b)**. The model was built and then the smart algorithm in the Forcite module was applied to optimise its geometry and the resulting model was named SiO_2-OH.

In order to study the wettability of the surface of octadecyltriethoxysilane-modified diatomaceous earth, a model of the product of hydrolysed octadecyltriethoxysilane was built as shown in **Figure S1(a)**, and then the geometry was optimised using the smart algorithm in the Forcite module. The hydrolysed product of octadecyltriethoxysilane with one hydroxyl group removed was connected to the surface of SiO_2-OH, with one hydrogen atom removed to obtain the model named SiO_2-OTS, as shown in **Figure S1(c)**.

Solid–liquid interface modelling

Solid–liquid models with different surfaces and different chemical environments were established. The Amorphous cell tool was used to construct the pure water box with the dimensions of a=b=c=49 Å and the number of water molecules is 3932; then the Build Layer tool was applied to distribute the pure water box on the SiO_2-OH surface and the SiO_2-OTS surface. Since the periodic boundary conditions may affect the interfaces, it is necessary to add a certain size of the water box above the of the vacuum layer; the established models are called SiO_2-OH/H_2O system and SiO_2-OTS/H_2O system, and the models are shown in **Figure S1(d, e).**

In this study, 1 mol/L HCl solution boxes and 1 mol/L NaOH solution boxes were constructed with dimensions a=b=c=49 Å. These solution boxes were combined with SiO_2-OH and SiO_2-OTS surfaces, which led to the solid–liquid interfacial models under different chemical environments. The constructed models were named SiO_2-OH/HCl solution system, SiO_2-OTS/HCl solution system, SiO_2-OH/NaOH solution system, and SiO_2-OTS/NaOH solution system, respectively.

After the model construction, we simulated the model using LAMMPS software. Firstly, a 30,000-step geometry optimisation was executed on the model to ensure the stability of the model structure. After the geometry optimisation process, the system was subjected to kinetic simulations at a temperature of 298 K for 1000 ps.

Dosage modulation of OTS-modified diatomaceous earths

Under alkaline conditions, OTS molecules firstly undergo a hydrolysis reaction to form CH_3-$(CH_2)_{17}$-Si-$(OH)_3$ and ethanol. Subsequently, the generated CH_3-$(CH_2)_{17}$-Si-$(OH)_3$ not only undergoes polycondensation itself but also reacts with the hydroxyl groups on the surface of the diatomaceous earth, a process that results in the surface of the diatomaceous earth being covered by a polysiloxane layer, which in turn reduces the surface energy [4]. Due to the formation of non-polar carbon long chains on the surface of diatomaceous earth, the interaction of water molecules with these long chains is weak, which makes the modified diatomaceous earth show hydrophobic characteristics.

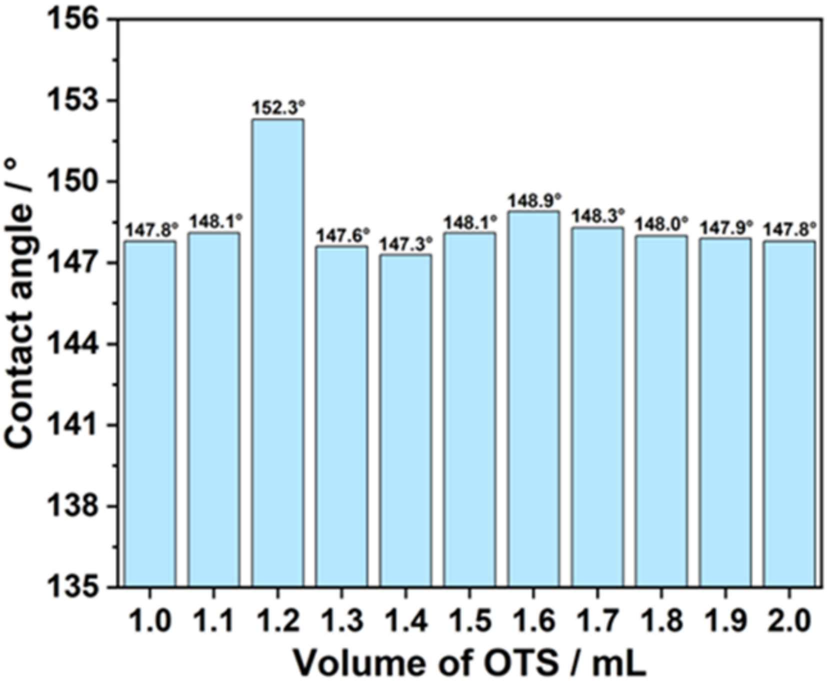

Figure S2. Relationship between OTS dosage and contact angle

With the increase in OTS dosage, the change in the contact angle of the coating showed a trend of decreasing, increasing, and then decreasing. When the OTS dosage was 1.2 mL, the coating exhibited optimal hydrophobicity, and the contact angle reached 152.3° (±0.6°). The increase in OTS concentration initially led to an increase in the contact angle, but when the concentration exceeded a certain threshold, the continued increase in the OTS concentration instead led to a decrease in the contact angle. The hydrolysis process of octadecyltriethoxysilane follows one-stage or pseudo-stage reaction kinetics within a certain concentration range, and the hydrolysis rate is accelerated with the increase in OTS concentration, and the corresponding amount of CH_3-$(CH_2)_{17}$-Si-$(OH)_3$ generated increases. However, when the OTS concentration reaches a certain limit, the hydrolysis rate decreases instead, leading to a decrease in the amount of CH_3-$(CH_2)_{17}$-Si-$(OH)_3$ generated, which in turn reduces

the number of molecules that undergo condensation reactions with the diatomaceous earth surface, and ultimately results in a decrease in the contact angle of the modified diatomaceous earth.

Molecular dynamics study of surface wettability of SiO_2-OH and SiO_2-OTS

Effect of hydrogen bonding on surface wettability

The formation of hydrogen bonds has a significant effect on the wettability of solid surfaces. In order to investigate this phenomenon, we study the generation of hydrogen bonds between solid surfaces and water molecules in two different systems by calculating the radial distribution function (RDF). The RDF is a method that describes the ratio of the density of particles at a specific distance with respect to a reference particle in a certain volume to the average particle density of the system, and its mathematical expression is given as follows. The peak position of the RDF reveals the interaction strength: the lower the peak position and the higher the peak, the stronger the interaction. Hydrogen bonding is a special type of inter- or intramolecular interaction that usually occurs when a hydrogen atom forms a covalent bond with an atom X with a large electronegativity and then approaches another atom Y with a large electronegativity and a small radius, forming the structure X-H...Y.

As **shown in Figure S3(a)**, we demonstrate the RDF between the H atoms in the surface hydroxyl group and the O atoms in the water molecule in the SiO_2-OH system, and between the H atoms in the surface CH_3-$(CH_2)_{17}$-$Si(OH)_2$-group and the O atoms in the water molecule in the SiO_2-OTS system. The SiO_2-OH system shows a

significant peak at r = 3.01 Å, which indicates the formation of hydrogen bonds. On the contrary, the RDF of the SiO_2-OTS system does not show a significant peak in the same range, implying that hydrogen bonding is not formed. This indicates that the H atoms in the surface hydroxyl groups are able to form hydrogen bonds with the O atoms of the water molecules, whereas the H atoms in the CH_3-$(CH_2)_{17}$-$Si(OH)_2$-group are unable to form hydrogen bonds with the O atoms of the water molecules. The presence of hydrogen bonds enhances the adsorption of water molecules on the solid surface, which in turn affects the wettability properties of the SiO_2-OH surface and the SiO_2-OTS surface. The inability of the H atoms in the CH_3-$(CH_2)_{17}$-Si- groups on the SiO_2-OTS surface to form hydrogen bonds with the O atoms of the water molecules decreases the number of hydrogen bonds in the SiO_2-OTS system, which in turn improves the material's hydrophobicity.

Table S1 shows the classification of different atom types under the CVFF, which may differ even if the elements are the same if the atoms to which they are attached are different. As shown in **Figure S3(d)**, the SiO_2-OH surface is rich in hydroxyl groups, and these hydroxyl groups are able to form hydrogen bonds with water molecules, thus enhancing the adsorption of water molecules on the surface. In contrast, on the SiO_2-OTS surface at equilibrium, as shown in **Figure S3(e)**, the CH_3-$(CH_2)_{17}$-$Si(OH)_2$-group transforms from an initially upright conformation to a flat-laying state, and this transition leads to a decrease in the number of hydroxyl groups exposed on the surface.

In the SiO_2-OH/H_2O system, the number of hydrogen bonds was 4631, whereas

in the SiO_2-OTS/H_2O system, the number of hydrogen bonds was reduced to 3159, indicating a significant decrease in the number of hydrogen bonds on the hydrophobic surface. Due to the decrease in the number of exposed hydroxyl groups on the surface, the wettability of the surface is weakened accordingly, which effectively enhances the hydrophobicity of the material.

Table S1. Atom types in the CVFF.

element of a set	atomic type	descriptions
O	oh	Oxygen atoms bound to H
O	o	sp^3 hybridised oxygen atom
O	oz	Oxygen atoms in zeolites or silicates
O	o*	Oxygen atoms in water molecules
Si	si	silicon atom
Si	sz	Silicon atoms in zeolites or silicates
H	ho	Hydrogen atoms bound to O
H	h	Hydrogen atoms bonded to C, Si or H
H	h*	Hydrogen atoms in water molecules
C	c2	sp^3 carbon bound to 2 H, 2 heavy atoms
C	c3	sp^3 carbon in methyl (CH_3-) groups

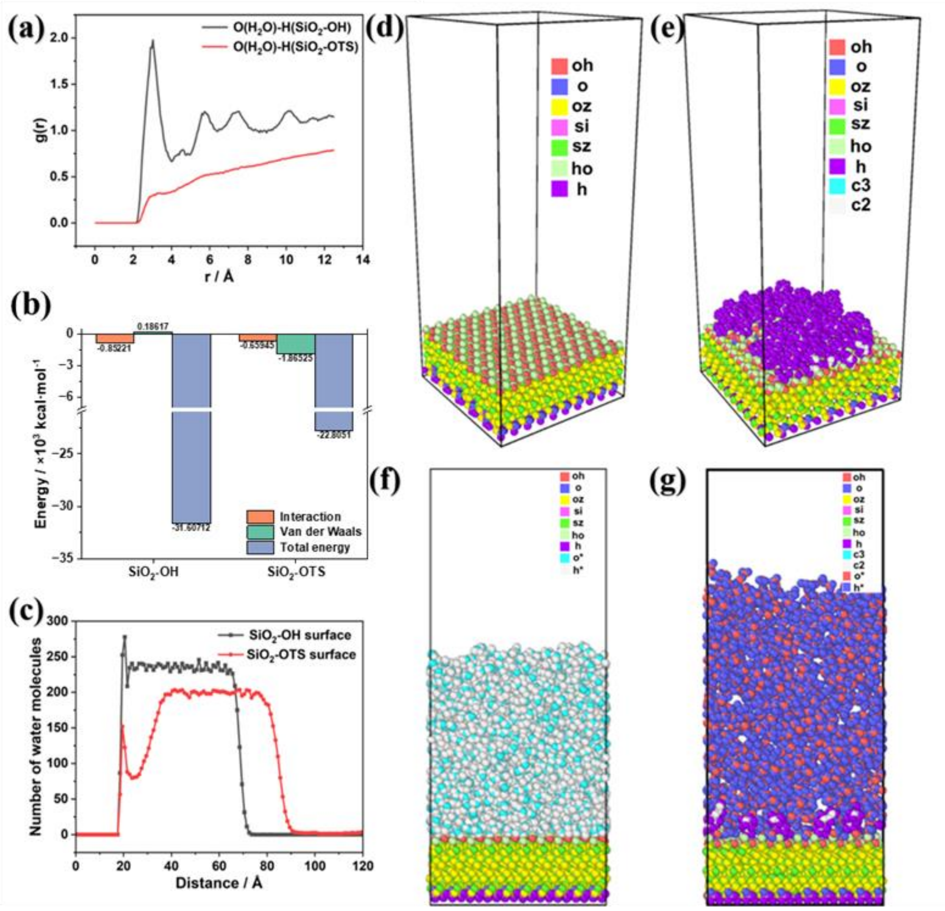

Figure S3. (a) Radial distribution functions of H and O atoms in SiO_2-OH and SiO_2-OTS systems; (b) interactions with water, van der Waals effect, and total energy of the system at the SiO_2-OH and SiO_2-OTS surfaces; (c) Z-direction distributions of water molecules at the SiO_2-OH and SiO_2-OTS surfaces; (d) equilibrium configuration on the SiO_2-OH surface; (e) equilibrium configuration on the SiO_2-OTS surface; (f) equilibrium configuration of water on the SiO_2-OH surface; (g) equilibrium configuration of water on the SiO_2-OHS surface.

Effect of solid–liquid interaction on wettability

Solid–liquid interfacial interaction is a key factor in the measurement of surface wettability: the stronger the interaction, the better the wettability of the solid surface. The solid–liquid interaction energy can be calculated by the following equation: $E_{interaction} = E_{total-} (E_{liquid} + E_{solid})$. Where $E_{interaction}$ represents the solid–liquid interaction

energy, E_{total} is the total energy of the system, E_{liquid} is the energy of the liquid phase, and E_{solid} is the energy of the solid surface.

Figure S3(b) demonstrates the interaction energy of SiO_2-OH and SiO_2-OTS systems with water molecules. The results show that the SiO_2-OH system has a lower interaction energy (-852.21 kcal/mol), indicating that water molecules are more likely to adsorb on its surface and exhibit better wettability. Whereas, the SiO_2-OTS system has a higher interaction energy (-659.45 kcal/mol), indicating a more hydrophobic surface. In the SiO_2-OH and SiO_2-OTS systems, the van der Waals energy of the SiO_2-OH system is 186.17 kcal/mol, while that of the SiO_2-OTS system is lower, at -1865.25 kcal/mol. The change in surface groups leads to a change in van der Waals forces. Calculation results indicate that the covalent modification of silica surfaces by octadecyl trichlorosilane (OTS) significantly increases the total system energy in an aqueous environment by approximately 8802 kcal/mol. This substantial energy increase stems from the strong hydrophobic effect triggered by the introduction of hydrophobic alkyl chains, theoretically confirming that OTS modification is the key driving force inducing the surface to transition from hydrophilic to hydrophobic.

Distribution of water molecules on SiO_2-OH, SiO_2-OTS surfaces

Figure S3 (f, g) demonstrates the distribution of water molecules on the SiO_2-OH versus SiO_2-OTS surfaces. The difference in water molecule distribution between the two surfaces at equilibrium is obvious. Water molecules on the SiO_2-OH surface are tightly adsorbed and show hydrophilicity, whereas water molecules on the SiO_2-OTS surface are widely spaced and show hydrophobicity. The hydrophobic surface

water molecules diffuse farther, indicating that the CH_3-$(CH_2)_{17}$-$Si(OH)_2$-group significantly increases the hydrophobicity of SiO_2-OTS.

Figure S3(c) demonstrates the z-axis direction distribution of water molecules on the SiO_2-OH and SiO_2-OTS surfaces. We define the region from the SiO_2 surface (z = 17.5 Å) to z = 21.5 Å as the adsorption layer of water molecules on the surface [5]. In this region, we can observe that the number of water molecules on the SiO_2-OH surface stays high and smooth, while the number of water molecules on the SiO_2-OTS surface rises and then levels off. In particular, the number of water molecules on the SiO_2-OH surface peaks at z = 20.5 Å, which is significantly higher than that on the SiO_2-OTS surface. This distribution feature indicates that the SiO_2-OH surface is able to adsorb more water molecules and shows stronger hydrophilicity, while the SiO_2-OTS surface shows better hydrophobicity due to the fact that the water molecules diffuse farther and adsorb relatively less. This phenomenon is closely related to the number of hydrogen bonds and solid–liquid interfacial interactions on the surface, which is in agreement with the previous calculations.

Hydrophobicity of Dia/OTS/EP materials

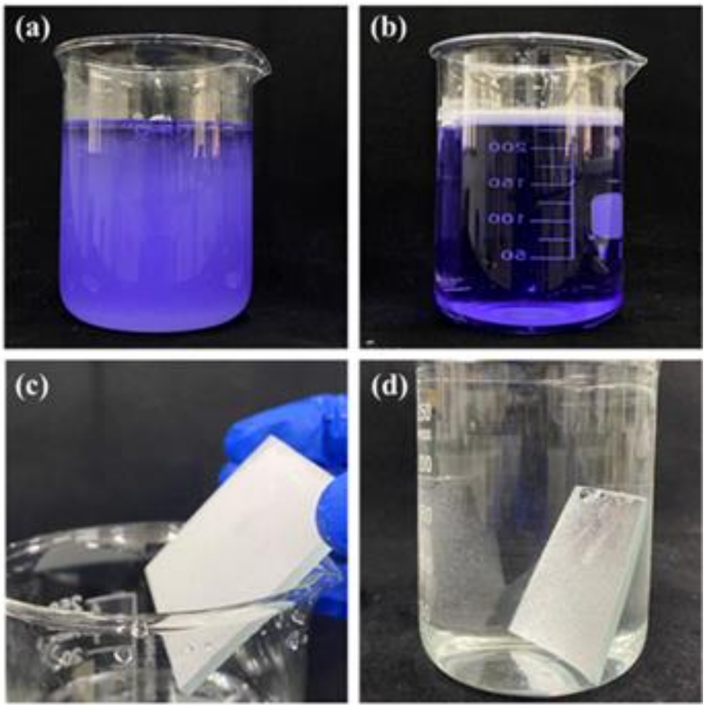

Figure S4. (a) Dispersion test of Dia in water; (b) Dispersion test of Dia/OTS powder in water; (c) Physical image of Dia/OTS/EP coating before immersion in water; (d) Physical image of Dia/OTS/EP coating immersed in water.

Figure S4(a, b) demonstrates the dispersion of diatomite in water before and after modification. Unmodified diatomaceous earth was uniformly dispersed in methyl blue water after stirring and precipitated after standing, showing good hydrophilicity due to its hydrophilic hydroxyl-rich surface. In contrast, Dia/OTS powder is not easily soluble in polar aqueous solutions due to its surface covered with a layer of polysiloxane, whose carbon long chains have non-polar properties. **Figure S4(c, d)** demonstrates the changes in Dia/OTS/EP coating before and after water immersion. The coating is white when it is not in contact with water and turns silver when it is immersed in water, which is due to the diatomaceous earth structure on the surface of the coating that forms micron-scale roughness and traps the air cushion to

form an air film, resulting in a silver sheen from the reflection of light in the water, demonstrating its excellent hydrophobicity.

Molecular dynamics calculation of chemical stability of Dia/OTS materials

Based on the previously established SiO_2-OTS model, we further constructed solid–liquid interfacial models of SiO_2-OTS in contact with pure water, 1 mol/L HCl solution, and 1 mol/L NaOH solution. Through molecular dynamics simulations, we calculated and analysed the changes in the distribution of water molecules, the changes in the diffusion behaviour of water molecules, and the changes in the total energy, van der Waals energy, and electrostatic interaction energy of the system on the SiO_2-OTS surface in the systems of pure water, 1 mol/L HCl solution, and 1 mol/L NaOH solution. These calculations provide an important basis for us to deeply investigate the chemical stability of Dia/OTS materials from a theoretical point of view.

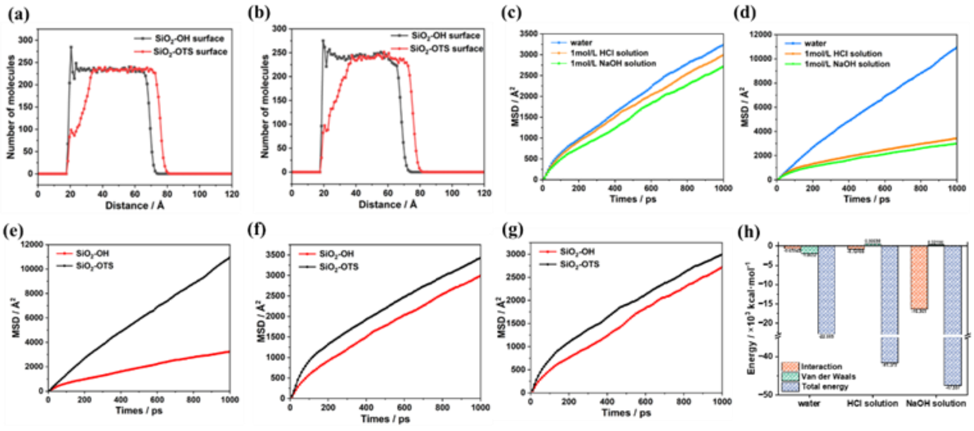

Figure S5. (a) Number of water molecules along the z-axis direction on the SiO_2-OTS surface in 1 mol/L HCl solution; (b) Distribution of water molecules along the z-axis direction on the SiO_2-OTS surface in 1 mol/L NaOH solution; (c) Mean-square displacements (MSDs) [1] on the SiO_2-

OH surface; (d) MSDs on the SiO_2-OTS surface; (e) Pure water in the water molecules on the SiO_2-OH surface and the SiO_2-OTS surface; (f) MSD of water molecules in 1 mol/L HCl solution on the SiO_2-OH surface and the SiO_2-OTS surface; (g) MSD of water molecules in 1 mol/L NaOH solution on the SiO_2-OH surface and the SiO_2-OTS surface; (h) MSD of different solutions with the SiO_2-OTS solid–liquid interactions, van der Waals, and total energy of the system.

Distribution of water molecules on the SiO_2-OTS surface in solutions with different pHs

Figure S5(a, b) shows the distribution of the number of water molecules on the SiO_2-OTS surface along the z-direction for different pH solutions. The number of water molecules above the adsorption layer gradually increases with the increase of z-axis value and finally reaches a steady state. However, in the pure water system, the stabilised values of the number of water molecules were significantly lower than those in the acidic and alkaline environments (183, 234, and 241 in the pure water, acid, and alkaline systems, respectively). Moreover, in the pure water system, the number of water molecules gradually tends to zero after diffusion up to 91.5 Å, whereas in the acidic and alkaline systems, the number of water molecules begins to converge to zero after diffusion up to 81.5 Å. The number of water molecules in the acidic and alkaline systems is significantly lower than that in the acidic and alkaline environments.

Accordingly, we can infer that both acidic and alkaline environments affect the hydrophobicity of SiO_2-OTS surfaces. The number of water molecules adsorbed on the SiO_2-OTS surface in acidic and alkaline solutions exceeds that in pure water, and the diffusion distance of water molecules is shortened.

Diffusion of water molecules on the SiO_2-OTS surface in different pH solutions

Figure S5(e–g) demonstrates the mean square displacement (MSD) of water molecules on the SiO_2-OH and SiO_2-OTS surfaces at different pH values. **Figure S5(c, d)** shows the variation in MSD of water molecules under these conditions. The results show that the diffusion coefficient of water molecules on the SiO_2-OTS surface is always higher than that on the SiO_2-OH surface regardless of the solution environment, indicating that the SiO_2-OTS surface remains hydrophobic under different chemical environments. This is consistent with the previously measured contact angle data and verifies the consistency of the experimental observations. Comprehensively analysing the data in **Figure S5(c–g)**, we calculated the diffusion coefficients of water molecules on the SiO_2-OH and SiO_2-OTS surfaces in different solutions, and the results are listed in **Table S2**. The acidity and alkalinity affect the diffusion of water molecules, and the larger the diffusion coefficient is, the faster the migration rate is. On both surfaces, the diffusion coefficient of water molecules was the lowest in NaOH solution and the highest in pure water. The SiO_2-OTS surface showed the largest increase in diffusion coefficient in pure water and the smallest in NaOH solution, suggesting that water molecules migrate the slowest in the alkaline solution, which relatively attenuates the hydrophobicity of SiO_2-OTS.

Table S2. diffusion coefficients of water molecules in different systems (cm^{-5}/s)

systems	diffusion coefficient
SiO_2-OH/H_2O	0.5025
SiO_2-OTS/H_2O	1.7578

SiO_2-OH/HCl solution	0.4594
SiO_2-OTS/HCl solution	0.4863
SiO_2-OH/NaOH solution	0.4287
SiO_2-OTS/NaOH solution	0.4382

Effect of pH on the interaction of SiO_2-OTS system

Figure S5(h) demonstrates the interaction energy analysis between the SiO_2-OTS surface and water molecules under different solution conditions. We can observe that the surface–water molecule interaction is most significant in 1 mol/L NaOH solution. This strong interaction leads to enhanced adsorption of water molecules on the SiO_2-OTS surface, which affects its hydrophobicity and makes it relatively weaker. In contrast, the interaction energy in 1 mol/L HCl solution was lower, indicating that the hydrophobicity of the SiO_2-OTS surface under this condition, although slightly inferior to that of the pure water system, was still superior to the performance in NaOH solution. This finding further supports the trend revealed by previous experiments that the nature of the solution affects the hydrophobic behaviour of solid surfaces. This is because the ions present in the electrolyte solution effectively disrupt the highly ordered water molecule structure near the hydrophobic interface. Na^+ in NaOH solution and Cl^- in HCl solution migrate to the hydrophobic interface, disrupting the hydrogen bond network, increasing the entropy in the interface region, and thereby significantly reducing the system's free energy. Additionally, under alkaline conditions, the SiO_2 substrate surface may carry more negative charges (Si-O^-), further attracting Na^+ ions to form a hydrophilic double

layer, resulting in a smaller contact angle in alkaline solutions.

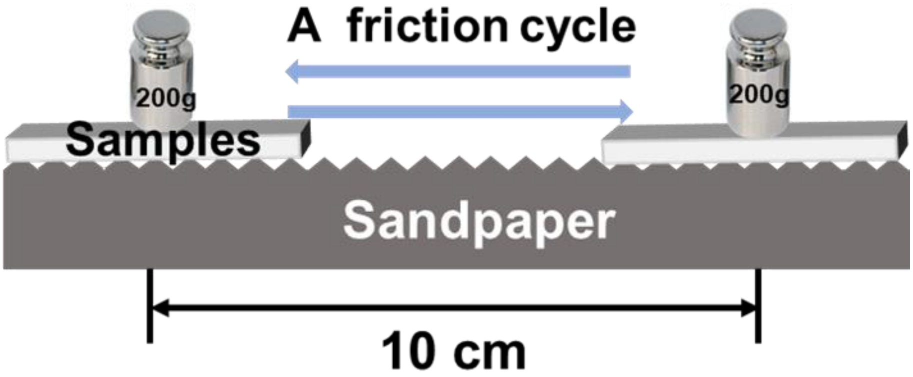

Figure S6. Schematic of sandpaper abrasion experiment

[1] H. Wei, J. Feng, C. Ma, Z. Li, M. He, J. Wang, X. You, L. Li , Effect of iron doping on the hydrophobicity of titanium dioxide film: experiment and simulation, J. Molecular Physics. 118 (2020) 1-9. https://doi.org/10.1080/00268976.2019.1696477

[2] Y.L. Yaphary, Z. Yu, R.H.W. Lam, D. Hui, D. Lau, Molecular dynamics simulations on adhesion of epoxy-silica interface in salt environment, J. Composites Part B. 131 (2017) 165-172. https://doi.org/10.1016/j.compositesb.2017.07.038

[3] X. He, T. Lou, P. Cao, X. Bai, C. Yuan, C. Wang, A. Neville, Experimental and molecular dynamics simulation study of chemically stable superhydrophobic surfaces, J. Surface & Coatings Technology. 418 (2021) 127236. https://doi.org/10.1016/j.surfcoat.2021.127236

[4] A. A. Issa, M. El-Azazy, A. S. Luyt, Kinetics of alkoxysilanes hydrolysis: An empirical approach, J. Scientific reports. 9 (2019) 17624. https://doi.org/10.1038/s41598-019-54095-0

[5] W. Zheng, C. Sun, B. Bai, Molecular Dynamics Study on the Effect of Surface Hydroxyl Groups on Three-Phase Wettability in Oil-Water-Graphite Systems, J. Polymers. 9 (2017) 370. https://doi.org/10.3390/polym9080370
